# Optical probes of molecules as nano-mechanical switches

**DOI:** 10.1038/s41467-020-19703-y

**Published:** 2020-11-20

**Authors:** Dean Kos, Giuliana Di Martino, Alexandra Boehmke, Bart de Nijs, Dénes Berta, Tamás Földes, Sara Sangtarash, Edina Rosta, Hatef Sadeghi, Jeremy J. Baumberg

**Affiliations:** 1 NanoPhotonics Centre, Cavendish Laboratory, University of Cambridge, Cambridge, CB3 0HE UK; 2 Department of Chemistry, King’s College London, London, SE1 1DB UK; 3 School of Engineering, University of Warwick, Coventry, CV4 7AL UK; 4 Department of Physics and Astronomy, University College London, London, WC1E 6BT UK

**Keywords:** Molecular electronics, Nanophotonics and plasmonics, Nanoparticles, Nanocavities, Nanophotonics and plasmonics

## Abstract

Molecular electronics promises a new generation of ultralow-energy information technologies, based around functional molecular junctions. Here, we report optical probing that exploits a gold nanoparticle in a plasmonic nanocavity geometry used as one terminal of a well-defined molecular junction, deposited as a self-assembled molecular monolayer on flat gold. A conductive transparent cantilever electrically contacts individual nanoparticles while maintaining optical access to the molecular junction. Optical readout of molecular structure in the junction reveals ultralow-energy switching of ∼50 zJ, from a nano-electromechanical torsion spring at the single molecule level. Real-time Raman measurements show these electronic device characteristics are directly affected by this molecular torsion, which can be explained using a simple circuit model based on junction capacitances, confirmed by density functional theory calculations. This nanomechanical degree of freedom is normally invisible and ignored in electrical transport measurements but is vital to the design and exploitation of molecules as quantum-coherent electronic nanodevices.

## Abstract (web summary)

The development of molecular electronics at single molecule level calls for new tools beyond electrical characterisation. Kos et al. show an optical probe of molecular junctions in a plasmonic nanocavity geometry, which supports in situ interrogation of molecular configurations.

## Introduction

**Fig. 1:**
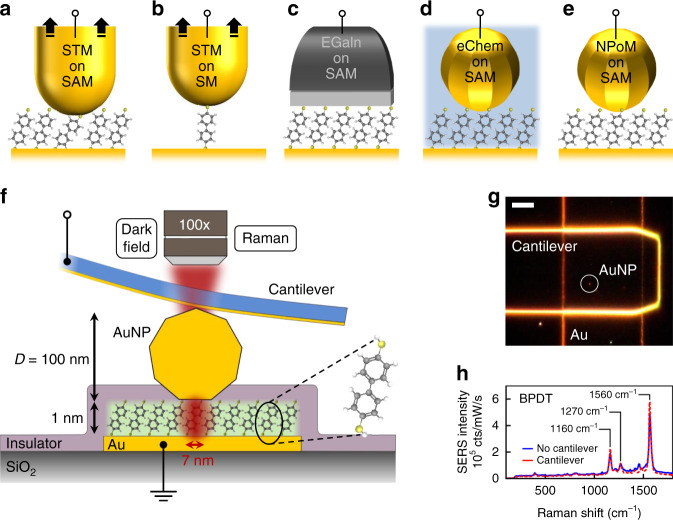
Schemes for contacting molecular junctions. **a**, **b** Junctions formed by scanning tunnelling microscope (STM) tip pull-off from **a** SAMs or **b** single molecules. **c** Eutectic GaIn alloy contacts. **d** Large area electrochemical cells indirectly contact AuNPs on SAMs and **e** scheme here using single plasmonic nanoparticle contact. **f**, **g** Detail of Au nanoparticle on SAM (NPoM geometry) which is electrically contacted by a conductive transparent cantilever, giving real-time optical access to the junction. Scale bar in **g** is 10 µm. **h** Raman spectroscopy of BPDT molecular layer in NPoM through the cantilever shows molecules are unperturbed. Laser power at sample is 0.2 mW, $\lambda$ = 633 nm, focussed to sub-μm spot.

Constructing electronic junctions with molecules is a potential avenue to achieve new device functionality, further junction miniaturisation and reduce energy consumption^1–4^. Metal-molecule-metal junctions have been extensively studied using scanning probe techniques and break junctions, typically through statistical analysis of electrical conductance in repeated transient pull-off experiments which shed little light on individual junction morphologies. Experiments which contact self-assembled molecular monolayers (SAMs) by pulling away a sharp tip^5–7^, until one final molecule bridges the tip-surface gap (Fig. 1a, b), probe different metal atomic configurations and molecular binding sites at each realisation^8,9^. Combining many thousands of such pull-off conductance measurements delivers systematic results which depend on the molecular electronic states. Alternative in situ averaging over many molecules through large-area contacts can also access molecular electronic properties^10–12^, with damage minimised by using nano-particulate or liquid metal contacts^13–15^ (Fig. 1c). Inferring the exact configuration of molecules in situ from these current-voltage measurements however is not easy, because the integrated device geometries prevent external probing of the junction during operation. Optical spectroscopies with the capability to follow molecular charging have been attempted by indirectly contacting molecules inside electrochemical cells^16,17^ (Fig. 1d), but suffer interference from ions in solution^18^ while probing regions with >10^15^ molecules.

In our devices, molecular monolayers are deposited on a flat Au substrate with single Au nanoparticles used as the top electrode, where the junction area can be scaled by using different diameters *D* (Fig. 1e, f). The extreme plasmonic coupling between nanoparticle and Au surface for such nm gaps allows in situ optical access to the behaviour of junctions under bias. Our measurements show that the molecules undergo conformational changes in the *V* < 1 V tunnelling regime. This new molecular nanomechanical mechanism shrinks MEMS functionality from the micro- to nano-domain.

This scheme exploits the tight light confinement inside *d* = 1–2 nm gaps (lateral optical full-width $\sqrt {Dd} \sim$ 7 nm) within nanoparticle-on-mirror (NPoM) structures^17,19^ to optically investigate ~100 junction molecules (Fig. 1f, g). Solution-deposited low-density *D* = 100 nm Au nanoparticles (AuNP) are conformally coated with an insulating parylene layer, and dry-etched to expose the AuNP crown (Methods section). NPoMs are individually contacted using a conductive transparent Si_3_N_4_ tip-less cantilever, giving negligible contact resistance (Supplementary Note 4).

Each NPoM is optically accessed through the cantilever for imaging and spectroscopy in real time during electrical measurements. The enhanced (*E* > 500) optical field within the NPoM plasmonic gap enables strong surface enhanced Raman spectroscopy (SERS ∝ *E*^4^) of the molecular spacer^17^, giving >100 kcounts/mW/s thus allowing short integration times for tracking dynamics. The collected Raman from the *λ* = 633 nm focussed laser (dashed Fig. 1h) originates only from molecules underneath the AuNP facet, initially biphenyl-dithiol (BPDT), showing their Raman-active vibrational modes which remain unchanged when contacted (Fig. 1h). Biphenyl SAMs are widespread in molecular electronics, known to form closely-packed uniform SAMs^20^ (giving consistent SERS), and provide large Raman cross-sections.

Changes in refractive index, thickness or conductivity of the molecular spacer can be tracked in real time through dark-field (DF) scattering spectroscopy of the electrically contacted NPoMs (seen red in circled DF image, Fig. 1g). Dark-field spectra of individual NPoMs with BPDT (Supplementary Fig. 2) are dominated by a strong peak at ∼680 nm from coupled plasmon oscillations between the AuNP and underlying Au mirror (Fig. 1f). Conductance measurements show that ∼50 molecules are contacted (Supplementary Note 4), while optical probing tracks a similar number of molecules under the facet^17^.

Using these optically accessible and reconfigurable but non-disruptive contacts for molecular electrical junctions we reveal that molecules twist under bias voltage, modifying their conductance. The applied bias triggers a redistribution of charge and potential across the junction, and molecules react by changing their conformation to minimise the overall dipolar and capacitive energies. This change is largely invisible in the electrical response, and only revealed by direct optical access to the molecules in the junction.

## Results

### Twisting of molecular rings upon applied bias

**Fig. 2:**
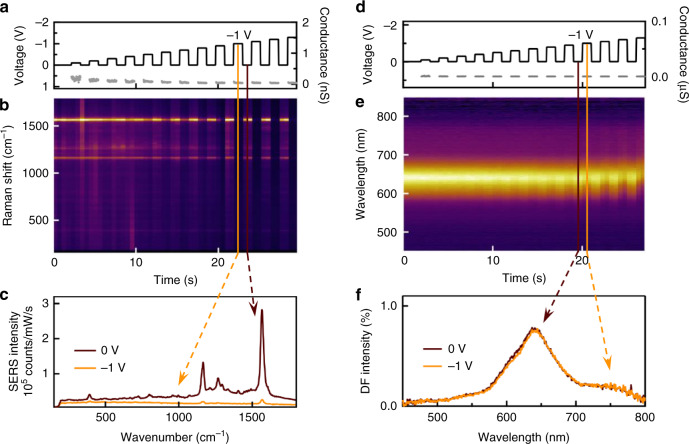
Raman (SERS) and dark-field switching under bias in single junction of BPDT. **a**, **b** Real-time SERS for increasingly negative bias voltage. **c** SERS spectra decrease 10-fold by −1 V bias. **d**–**f** Dark-field scattering intensity under bias, with decrease in amplitude and coupled plasmon redshift for $|V|$ > 1 V but **f** no change for −1 V bias.

The cantilever used to approach an individual NPoM junction is lowered until flat and parallel to the surface (Supplementary Fig. 1) to make an ohmic contact (Methods section), avoiding significant forces by bending (evidenced by the absence of vibrational Raman shifts from the 50 gap molecules probed). Electrical measurements are performed simultaneously with continuous Raman or dark-field spectral acquisitions. A constant voltage during each acquisition (Fig. 2a) gives highly repeatable spectra (Supplementary Figs. 4 and 5, molecules absorb only in UV thus giving no photocurrent). Strong reduction in SERS is seen whenever $\left| V \right|$ > 0.5 V, saturating above 1.0 V (Fig. 2b, c) with over 10-fold decrease. In dark-field no changes are detected for $\left| V \right|$ < 1 V (Fig. 2d–f). The collapse in SERS strength by $\left| V \right|$  = 0.5 V occurs before any significant variation in scattering spectrum (Fig. 2f), showing cantilever artefacts cannot be responsible. No effects are seen when the SAM is not included in the device. The conductance *G* < 1 nS for $\left| V \right|$ < 1.5 V remains in the linear direct tunnelling regime *G* < 10^−4^ *G*_0_, becoming nonlinear only for higher bias (Fig. 2a, d) where non-reversible redshifts of the NPoM coupled mode are seen (saturating at Δλ ∼ 80 nm). Similar effects are seen with both positive and negative bias (Supplementary Fig. 4).

Although the light intensity in the nano-gap does not change (shown by unchanging dark-field spectra), the 20-fold decrease in SERS shows that the molecular Raman cross-sections reduce (at slightly different rates, Supplementary Fig. 4). Biphenyl molecules have a delocalised *π* electron distribution^21^ due to near-alignment of the *π* orbitals across the C atoms connecting the two rings. Twisting of *π*-orbitals across this C–C link disrupts the delocalisation, reducing the molecule polarizability. Previous work however suggested that bias charges these molecules, which aligns the two rings and thus leads to an increased Raman cross section and a shift in the peak positions, as opposed to the decrease in signal observed here with no peak shifts (Supplementary Note 10)^16,22^.

**Fig. 3:**
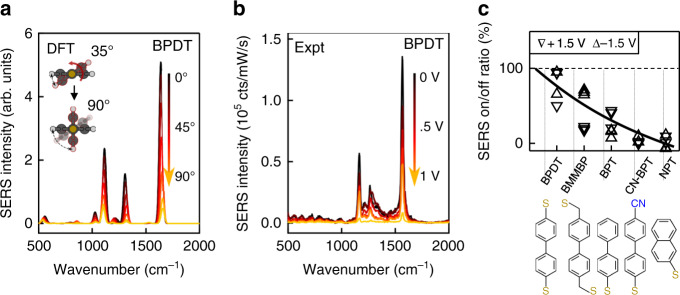
Comparison of Raman vs twist *θ* in theory and experiment. **a** DFT calculations show decrease in Raman intensity with increasing dihedral angle for BPDT. **b** Experimental reduction in SERS intensity as voltage increases from 0 V to 1 V. **c** SERS intensity reduction quantified as ratio at ±1.5 V vs 0 V for different biphenyl molecules and NPT (using Raman peak intensity at 1590 cm^−1^, 1070 cm^−1^ for NPT). Line shows model predictions.

We explore this twisting through density functional theory (DFT) on a BPDT molecule bound to Au atoms at both thiol terminal groups. The molecule is progressively twisted by changing the dihedral angle *θ* between the two ring planes from 0° (in-plane) to 90° (rings perpendicular to each other). The energy $U_{{\mathrm{DFT}}}(\theta )$ and Raman signal intensity are computed for each configuration (Methods section). The energy minimum at $\theta \sim 35^\circ$ sets the initial molecular state within the junction. The simulated Raman intensity decreases with increasing twist angle (Fig. 3a), and is minimised at 90° because the extended dipole across both rings is broken^23^ decreasing the Raman cross-section.

Our experiments reproduce this Raman suppression with increasing voltage (Fig. 3b), suggesting we indeed observe molecular twisting. This is supported by experiments on 2-naphthalene-thiol, which cannot twist and gives no voltage dependence (NPT in Fig. 3c, and Supplementary Fig. 5). Other biphenyl molecules with different functional groups still show SERS switching, but with decreased on/off SERS ratios and larger voltage thresholds when only one thiol group is present (Fig. 3c and Supplementary Note 3). While the 20 nm wide facets of 100 nm AuNP nanogaps accommodate ∼100 molecules in a single junction^17^, we can also observe twist switching of individual molecules (Supplementary Fig. 8). Given the sub-ps lifetime of the torsional mode, molecular NEMS switching potentially accesses the THz regime, promising for devices. Modulating the applied voltage in our experiments gives both a low frequency contribution to the switching (∼10 kHz) alongside a high-frequency contribution which persists above 1 MHz.

### Origin of molecular twisting behaviour

Previous models for bi-phenyl systems considered redox effects^22^ that modify the Raman cross-sections (flattening the molecule upon reduction, as replicated in DFT here, Supplementary Note 10), or changes in binding of the terminal group with Au adatoms^24^, that cannot explain our results. Instead, to investigate the origin of molecular twisting in our system, we implement detailed non-equilibrium calculations based on DFT modelling of the junction under applied bias (see Methods section and Supplementary Note 11). These calculations show that local charges and electrostatic potential distribute differently across the gap region depending on molecular configuration, and that a twisted ring conformation is energetically favoured as bias increases.

**Fig. 4:**
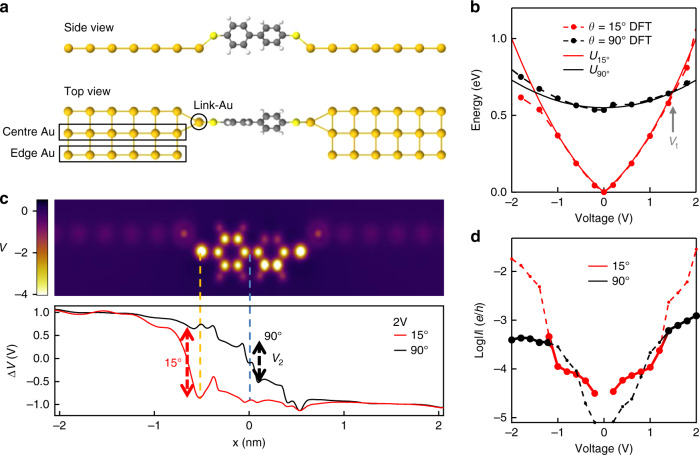
Modelling of junction under bias. **a** Model DFT geometry shown for $\theta$ = 15°, Au and S atoms remain fixed when changing $\theta$. **b** DFT energy for $\theta$ = 90° is lower than $\theta$ = 15° for $V$ > 1.5 V, while $U_{15^\circ }$, $U_{90^\circ }$ calculated from bond capacitances and dipoles (dashed) follow DFT, predicting configuration switch at $V_{\mathrm{t}}\sim$ 1.5 V. **c** Potential distributions in atoms at the molecule-lead interface for $\theta$ = 15°. Applied potential ${\mathrm{{\Delta}}}V$ drops predominantly across central C–C bond for $\theta$ = 90°, and across one Au–S interface for $\theta$ = 15°. **d** Calculated tunnelling between the leads, showing lack of twisting signature at $V_{\mathrm{t}}$.

The junction is modelled with a BPDT molecule bound to two identical Au leads via linker-Au atom protrusions (Fig. 4a), for fixed twist *θ* of the rings. Leads are one atom thick for faster convergence of calculations, but this agrees with the full three dimensional lattice of Au atoms (Supplementary Note 11). We calculate the energy of the system under bias for *θ* = 15° and *θ* = 90° (Fig. 4b), and observe that the energy of the *θ* = 90° configuration has a weaker voltage dependence, dropping below the *θ* = 15° energy for $V_{\mathrm{t}}$ > 1.5 V. To identify the origin of this trend, we extract the electrostatic potential profile along the junction including the leads and molecule in the two cases along with the Muliken charge distributions (Fig. 4c and Supplementary Fig. 19). In the *θ* = 90° case, around half of the applied potential is dropped across the central C–C bond (*V*_2_, black dashed arrow). From the charge stored vs potential drop across this bond, a capacitance $C_2$ = 0.015 aF is obtained, nearly independent of bias and matching that expected for a bond-sized capacitor (Supplementary Note 9). In a simple electrostatic picture, the total energy of this configuration is then

1 $$U_{90^\circ } = U_{{\mathrm{twist}}} + \frac{1}{2}C_2V^2$$

where $U_{{\mathrm{twist}}}$ = 0.55 eV is the energy required to twist the rings to *θ* = 90° (Supplementary Note 11). This model provides a surprisingly good account of the DFT calculation (Fig. 4b black dashed). By contrast for *θ* = 15° twists, the central C–C bond has higher conductance and gives negligible potential drop (Fig. 4c). Instead, the potential drop is almost entirely concentrated at one of the molecule-lead interfaces and is accompanied by a build-up of positive charge on the linker-Au and negative charge on the S. This decreases the dipole moment at the Au–S interface formed when the molecule binds. The same behaviour is reproduced at the opposite interface when the bias polarity is inverted (Supplementary Fig. 19). The total energy $U_{15^\circ }$ is then the sum of a linear contribution ${\mathrm{{\Delta}}}q\left| V \right|$, symmetric in voltage, given by the surface dipole induced by the field, and a quadratic capacitive term accounting for the charge on the S and surrounding Au atoms

2 $$U_{15^\circ } = {\mathrm{{\Delta}}}q\left| V \right| + \frac{1}{2}C_1V^2$$

with contact capacitance $C_1\sim$ 0.04 aF and ${\mathrm{{\Delta}}}q$ = 0.25*e* extracted from the DFT (Supplementary Note 11). Again this simplified $U_{15^\circ }$ accounts for the DFT energies well (Fig. 4b), in particular showing the low-field reversal of the induced surface dipole for opposite bias, supporting our interpretation based on local charges and potentials. The transition voltage $V_{\mathrm{t}}$ for triggering switching to *θ* = 90° when $U_{15^\circ } > U_{90^\circ }$ is then controlled by the relative contact energy

3 $$U_{\mathrm{c}} = {\mathrm{{\Delta}}}q.V_{\mathrm{c}} = \frac{1}{2}\frac{{{\mathrm{{\Delta}}}q^2}}{{\left( {C_1 - C_2} \right)}}\sim 0.20\,{\mathrm{eV}}$$

4 $$V_{\mathrm{t}} = 2V_{\mathrm{c}}\left\{ {\sqrt {1 + \frac{{U_{{\mathrm{twist}}}}}{{U_{\mathrm{c}}}}} - 1} \right\}\sim 1.5{\mathrm{V}}$$

close to the observed value of 1 V. This expression for $V_{\mathrm{t}}$ can be adapted to all molecules with a dihedral degree of freedom, where $U_{{\mathrm{twist}}}$ is modulated by side groups that determine the steric properties of the molecule, and $C_1 - C_2$ is set by the difference in capacitance between the twisting C–C bond and the molecule-lead interface, in turn related to metal type and molecular terminal group. The asymmetry of the current for negative and positive voltages (Fig. 4d) is due to asymmetry of the junctions, while we also observe a small rectification in our experiment (Supplementary Fig. 9). This is related to the asymmetry of the junctions imposed by the twisted biphenyl rings and their relative orientation to the Au–S–C angle. The energy profile changes slightly when the configuration to the electrodes changes (Supplementary Fig. 21), however the twisting behaviour is not affected.

### Circuit model of molecular junction

**Fig. 5:**
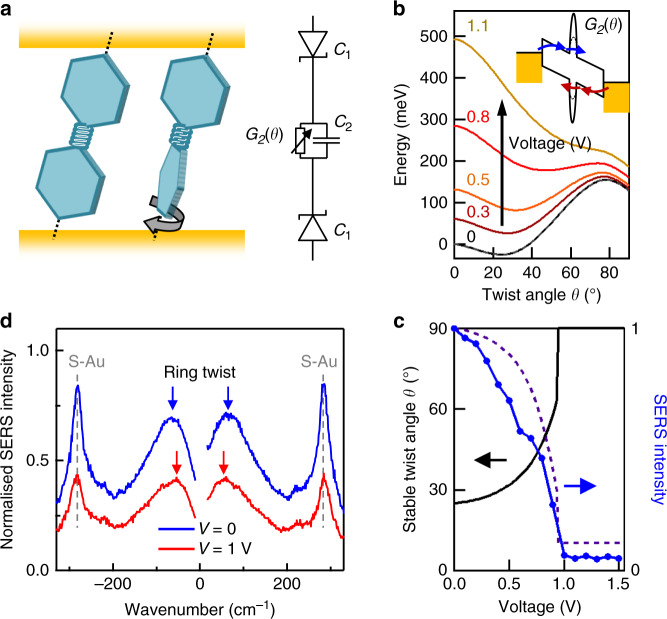
Circuit model compared to SERS. **a** Circuit model for BPDT molecules in the junction gap, with each section 1–3 of the junction characterised by its conductance and capacitance; central CC bond has variable conductance. **b** Calculated energy profile $U = U_{{\mathrm{DFT}}}\left( \theta \right) + U_{\mathrm{Q}}(\theta )$ as voltage is increased, shifting stable angle towards larger twists. **c** Voltage dependence of DFT twist angle $\theta _{{\mathrm{eq}}}\left( V \right)$ (black) and resulting SERS intensity (at 1590 cm^−1^) from DFT (dashed) compared to experiment (points). **d** Voltage-induced shift of central CC twist vibration to smaller wavenumbers (arrows). SERS normalised using a $T$ = 320 K thermal ratio between Stokes and anti-Stokes.

We translate the localised charges and potentials into an intuitive circuit model linked directly to the spectroscopic data. We divide the molecular junction into three sections, each with its own conductance and capacitance (Fig. 5a). Conductive AFM and STM break-junction experiments in liquid already showed that the tunnelling conductance $G_2$ through fixed-twist biphenyl molecules is controlled by *θ* as $G_2 = G_{{\mathrm{CC}}}(1 + g{\mathrm{cos}} ^{2}\theta )$ where $G_{{\mathrm{CC}}}\sim$ 76 μS and $g\sim$ 50 (refs. ^25–27^) (Supplementary Note 9). The molecule-Au contact regions are equivalent to Schottky diodes oriented back-to-back (Fig. 5a), so that when one is reverse biased the other is forward-biased (and thus conducting). The applied potential is then divided between the central bond (with variable barrier height depending on $\theta$) and the linker Au–S at the negative contact. Since the current $I = V_1G_1 = V_2G_2 = VG_t = (V_1 + V_2)\left[ G_1^{ - 1} + G_2^{ - 1} \right]^{ - 1}$, the electrostatic energy $U_{\mathrm{Q}} = \frac{1}{2}\left[ C_1V_1^2 + C_2V_2^2 \right] + {\Delta}q.V_1$ is simply obtained (noting $V_3\sim$ 0 from Fig. 4d; for negative bias $V_1$,$C_1$ and $V_3$,$C_3$ are swapped). The interface capacitances dominate the twist capacitance (Fig. 4e) because of the larger area at the contact. Adding the configuration energy $U_{{{{\mathrm{twist}}}}}(\theta )$ required to twist the molecule by an angle $\theta$ calculated from DFT, we obtain the total energy $U = U_{{\mathrm{twist}}}\left( \theta \right) + U_{\mathrm{Q}}(\theta )$. Plotting $U$ for different bias levels (Fig. 5b) indeed predicts that the stable twist angle $\theta _{{\mathrm{eq}}}\left( V \right)$ increases as voltage is applied, reaching 90° ~ 1 V.

The DFT Raman intensity at each $\theta _{{\mathrm{eq}}}\left( V \right)$ from Fig. 3a then predicts the SERS vs voltage, and matches our data well (Fig. 5c, see Supplementary Notes 9 and 11 for model details) providing a direct link between the experimentally observed modulation of SERS and the molecular twist. Crucially, although the switching is readily detected optically, in electrical transport we find that the full DFT calculations predict only smooth changes in conductance during switching (Fig. 4d and Supplementary Fig. 20), which is indeed observed (Supplementary Fig. 9). This therefore motivates the use of nano-optics in molecular electronics.

Inserting an extra carbon into the chain (BMMBP, Supplementary Note 3) reduces the molecular conductance $G_2$ which increases the voltage threshold for twisting, while removing the thiol from one end (BPT, CN-BPT) decreases the upper junction $C_1$, which also increases this threshold (Supplementary Note 9). Conduction in the |*V*| < 1 V regime is small (<nA, Fig. 2a, d) via direct tunnelling (Supplementary Note 9), but sets the potentials across each molecular section. At higher voltages significant currents flow, heating the junction and disrupting the molecular and Au structure resulting in DF spectral shifts^17,28^.

Our model is based on individual molecules, but cooperative twisting within the SAM may also be important^29^. The voltage-dependent torque, 61$V^2$ pN.nm, exceeds the DFT-estimated counter-torque of 35 pN.nm at a threshold $V_{\mathrm{t}}$ = 0.8 V in good agreement with experiment. The twisting energy per molecule, $U_{\mathrm{Q}}\sim 14k_BT\sim 56{\mathrm{zJ}}$ is robust to thermal fluctuations, despite being only 10x the lowest possible switching energy. Plasmonic nanocavities which allow us to extract Raman signatures, thus give in situ NEMS-based molecular torque measurements with a resolution of ~5 pN.nm.

To further confirm this molecular twisting, we directly observe the central C–C twist of BPDT at low wavenumbers (Methods section) in both Stokes and anti-Stokes SERS (Fig. 5d). The 0 V peak at 65 cm^−1^ corresponds well to the DFT potential $U_{{\mathrm{DFT}}}(\theta )$ (Fig. 5b). Compared to other Lorentzian SERS lines (<10 cm^−1^ FWHM), the C–C twist is a broad Gaussian (100 cm^−1^ FWHM), suggesting each molecule is sensitive to its subtly different molecular environment within the layer, which supports the idea that Fermi energy varies slightly across the molecules in the gap. As bias is applied, a 10-cm^−1^ shift to smaller wavenumbers of this C–C twist is seen, in agreement with the 27% reduction in curvature of the full potential $U(\theta )$ at 0.5 V in our circuit model (Fig. 5b) that should lead to $\sqrt {0.27}$ = 15% or 10 cm^−1^ reduction in vibrational energy. Adjacent SERS lines corresponding to the S–Au bond stretch show no detectable shift, while the Stokes: anti-Stokes ratio^17^ shows no change in temperature with bias. This nano-device measures real-time molecular twist angles, suggesting the capability for detecting binding events, for instance sensing trace-gas molecules.

## Discussion

We conclude that the energies obtained from the full quantum calculation of our molecular junction can be understood as being dominated by energies from electrostatics, and modelled as a classical electrical circuit based on surface dipoles and capacitance (Supplementary Fig. 21). Their energy cost as the bias increases drives a switch to the twisted molecular state. Surprisingly, we find that a dynamic conformational change in the molecule affects the coupling of the molecule itself to the leads, triggering a redistribution of charges and potential across the junction, in contrast with common views. Our findings demonstrate that the electrostatic potential profile changes significantly with bias voltage in a given molecular junction – a large potential drop at one interface between the planar BPDT molecule and gold electrode changes in the twisted BPDT to mostly develop across the C–C bond between the two rings (Fig. 4c). We also demonstrate that transport remains coherent at higher bias voltages and the molecule is not charged as suggested previously. This opens fascinating routes to utilise quantum interference at higher bias voltages for various nanoelectronic applications.

Our model emphasises that theories of molecular electronics treating leads and molecules as separate objects are incomplete, and inclusion of local potentials, charges, and interfaces is needed even for simple junctions. In the tunnelling regime here, the capacitive energy saved by dropping potential across the rings yields elastic potential energy to twist the molecular torsion spring. Compared to conformational changes in molecules from STM^22^, our direct contacting of NPoM structures is performed in ambient conditions and without feedback currents passing through the molecule for tip positioning, and much closer to realistic device configurations. Future work to access *ac* conductivities through smaller area cantilevers would be useful to determine circuit model parameters directly. Our versatile contacting technique can be applied to a wide variety of other nanostructures such as plasmonic dimers or metasurfaces. Our findings have critical implications in the design of novel devices based on quantum interference that rely on molecular conjugation, since the molecule-lead interactions can disrupt conjugation and modify device functionality. Promising molecules to explore include para vs meta OPE3, anthracene, anthraquinone and dihydropyrene derivatives. Coupling molecular twisting, tunnelling electronic transport and plasmonics may also lead to novel modes of light emission^30^.

## Methods

### Sample preparation

Device fabrication starts with 10 nm of Cr for adhesion followed by 100 nm of Au deposited by evaporation onto a 250-nm-thick SiO_2_ on Si substrate in a pattern defined using shadow-mask evaporation. Samples are left overnight in a 1-mM solution of the desired molecule (BPDT, BPT, BMMBP, CN-BPT and NPT) in ethanol to deposit a self-assembled monolayer, and are then rinsed with ethanol. Gold nanoparticles (AuNPs, 100 nm diameter) from BBI solutions are drop-cast on the surface to obtain a number density of ~0.001 µm^−2^ on the Au pattern for contacting individual AuNPs through the cantilever. Sample are post-coated with a ~350-nm thick uniform layer of parylene-C (SCS Labcoter 2) at room temperature, and the parylene-C layer is then selectively etched using O_2_ plasma to expose the top of the AuNPs while leaving ~20–30 nm insulating layer around the sides and base (see Supplementary Note 1).

### Conductive cantilever preparation

Transparent tip-less SiN AFM cantilevers (200 µm long, 35 µm wide and 600 nm thick) are coated with a 3-nm Cr adhesion layer and 6 nm Au by e-beam evaporation. The quality of the conductive film is verified with scanning electron microscopy and a uniform film without pinholes is obtained, with a sheet resistance 3Ω/square.

### Optical microscopy and spectroscopy

Optical dark-field images are recorded in a custom-modified Olympus BX51 microscope. Samples are illuminated with a focussed white light source (halogen lamp). The scattered light is collected through a ×100 dark-field objective (Olympus LMPLFLN100xBD, NA 0.8) and analysed with a fibre-coupled (50 µm core diameter optical fibre) Ocean Optics QE65000 cooled spectrometer. We use a standard diffuser as a reference to normalise white light scattering.

### Atomic force microscopy

Sample topography is performed using an Asylum Research MFP-3D atomic force microscope in non-contact AC mode. Asylum Research AFM Software version 15 is used for instrument control and data post-processing.

### Raman spectroscopy

Raman spectroscopy is performed in the same custom-modified Olympus BX51, using a 633-nm excitation laser with 200 µW power focussed into a diffraction limited spot. The Raman emission is detected after separation through a dichroic beamsplitter and laser line filter using an Andor grating monochromator and EMCCD. Low wavenumber measurements are performed with the same system using a 785 nm excitation laser with 200 µW power.

### DFT calculations

Computational spectra are calculated after geometry optimisation with B3LYP hybrid functional employing Grimme’s D3 dispersion correction with Becke-Johnson damping. The basis set of def2SVP was used including the pseudopotential definitions for gold atoms. Potential bias was modelled as a homogeneous dipole field. Raman intensities were recalculated from the polarizability derivatives according to the experimental setup, including the temperature and frequency of the excitation laser. Numerical integrals were computed on an ultrafine grid, as method implemented in Gaussian09 Rev. E (Supplementary Note 6).

### Non-equilibrium transport calculations using DFT under bias

To investigate the quantum transport through BPDT molecules between gold electrodes, we first carried out geometry optimisation to the force tolerance of 10 meV/Å using the *SIESTA* implementation of density functional theory (DFT), with a single-ζ basis set and the Local Density Approximation (LDA) functional with CA parameterisation. A real-space grid was defined with an equivalent energy cut-off of 250 Ry. We applied bias voltage under non-equilibrium conditions and calculated the Hamiltonian and electrostatic potential for each bias voltage. From the converged DFT calculation, the underlying mean-field Hamiltonian $H$ was combined with our quantum transport code, *Gollum* to calculate transmission coefficient $T(E,V_{\mathrm{b}})$ for electrons of energy *E* passing from the source to the drain. We then calculate the room-temperature current $I(V_{\mathrm{b}})$ from the obtained $T(E,V_{\mathrm{b}})$ using the Landauer formula $I(V_{\mathrm{b}}) = \frac{{2e}}{h}\mathop {\smallint }\nolimits_{\!\!- \infty }^{ + \infty } dET\left( {E,V_{\mathrm{b}}} \right)\left\{ {f\left( {E,T,V_{\mathrm{b}}/2} \right) - f\left( {E,T, - V_{\mathrm{b}}/2} \right)} \right\}$ (see Supplementary Note 11 for more details).

## Supplementary information

Supplementary Information

## Data Availability

Research data is available from the authors or at: 10.17863/CAM.59585.

## Code availability

References to software and code used for DFT calculations are provided in the Methods section.
