# Supplementary material for: Optical probes of molecules as nano-mechanical switches
Source: Nat Commun. 2020 Nov 20;11:5905. doi: 10.1038/s41467-020-19703-y (PMC7679449; doi:10.1038/s41467-020-19703-y)
Supplement: Supplementary file 1 — Supplementary Information [file 41467_2020_19703_MOESM1_ESM.pdf]

SUPPLEMENTARY INFORMATION

Optical Probes of Molecules as Nano-Mechanical Switches

Dean Kos[1], Giuliana Di Martino[1,*], Alexandra Boehmke[1], Bart de Nijs[1], Dénes Berta[2], Tamás Földes[2], Sara Sangtarash[3], Edina Rosta[4,*], Hatef Sadeghi[3,*], Jeremy J. Baumberg[1,*]

[1] NanoPhotonics Centre, Cavendish Laboratory, J J Thomson Avenue, University of Cambridge, CB3 0HE, UK

[2] Department of Chemistry, King's College London, London SE1 1DB, United Kingdom

[3] School of Engineering, University of Warwick, Coventry CV4 7AL, United Kingdom

[4] Department of Physics and Astronomy, University College London, London WC1E 6BT, United Kingdom

Supplementary Note 1. Device fabrication and cantilever control

A pattern of 10nm-Cr/100nm-Au lines of width 30µm is evaporated onto a SiO_2 substrate via shadow mask evaporation through a parallel-bar TEM grid (Agar Scientific), to make the bottom electrode (Supplementary Figure 1a). Avoiding lithography for this step maintains a cleaner surface free from photoresist or organic solvent contamination. This line pattern is used to reduce the electrode area when creating a cross junction upon approach with the transparent cantilever from the perpendicular direction (Supplementary Figure b), and prevents scratching off the sample layers by the front edge of the cantilever. The deposition of the SAM and AuNPs is done on the patterned Au electrodes as described in the Methods in the main text. Parylene is used as an insulating barrier to prevent contact between the cantilever surface and the bottom electrode, so molecular junctions are only produced through the AuNPs. Parylene is a common coating in the electronics industry[1], chosen here for the conformal coating properties and room temperature deposition, preserving the SAM. To achieve the insulating layer only around, and not on top of the AuNPs, a 350nm thick parylene film is first deposited on the entire device. This gives a near-smooth profile above the AuNPs, and the film is then isotropically dry etched in steps with O_2 plasma until the AuNP crown is exposed, leaving a 20-30nm thick parylene layer on everything else. Each etching step is monitored with AFM to achieve the correct device structure.

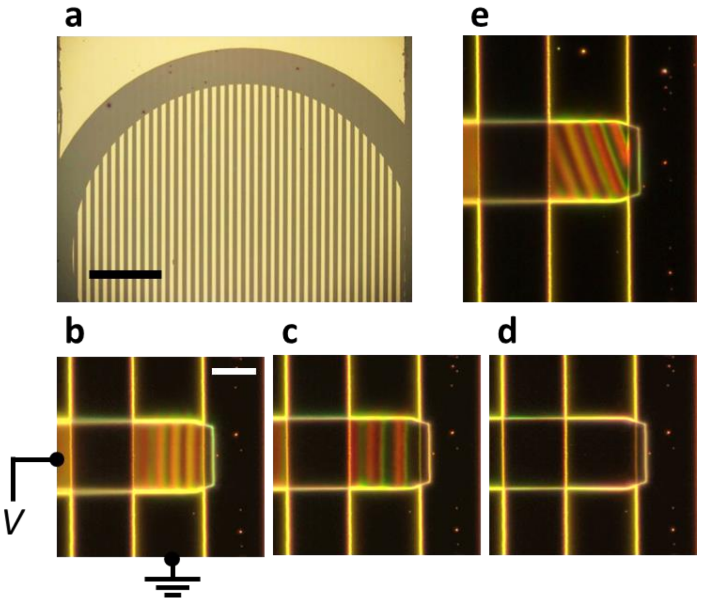

Supplementary Figure 1 | **a**, Evaporated Au pattern through parallel-bar TEM grid, scale bar is 500µm. **b**, Cantilever and electrode bias scheme, and wedge fringes upon first contact with the surface, **c**, intermediate position, and **d**, cantilever completely flat. **e**, Angled fringes for 1° cantilever tilt.

Cantilever position is controlled with an XYZ and rotation piezoelectric stage (SmarAct), with the cantilever mounted at 5° to the sample surface. As the cantilever is lowered along the Z direction, dense wedge interference fringes appear upon first contact with the surface. The fringe spacing progressively increases with further approach, until disappearing when the cantilever is flat and parallel to the surface (Supplementary Figure 1b-d). Vertical displacement of <100nm is easily monitored from the position and spacing of the fringes. Any cantilever tilt is also immediately detected from the angle of the fringes (Fig.S1e) and can be corrected to <0.2°. Reliable electrical measurements and spectroscopy on AuNPs are only possible when the cantilever is completely flat and parallel to the surface with no tilt. Introducing the cantilever on top of the NPoM maintains the peak shape, but blueshifts the coupled mode resonance by ~40nm from dielectric loading of the NP (Supplementary Note 2).

Supplementary Note 2. In-situ darkfield spectroscopy

The transparent cantilever affects darkfield spectroscopy of AuNPs because of the increased refractive index this introduces on top of the NPoM structure. We consistently observe a ~40nm blueshift in the resonance wavelength of the NPoM coupled mode, and a slight decrease in intensity of the single particle mode (Supplementary Figure 2).

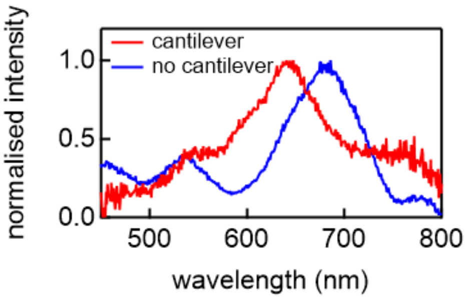

Supplementary Figure 2 | **Darkfield through cantilever**. Darkfield spectroscopy through the cantilever shows a blueshift in the NPoM coupled-mode peak.

The NPoM coupled mode remains clearly distinguishable in our *in-situ* measurements, and its evolution is tracked in real time with a stable signal (Supplementary Figure 3a). We observe no change in the amplitude or position of this peak in the tunnelling regime for $|V|$<1.5V, and voltage can be cycled repeatedly in this range for tens of minutes without detectable spectral changes. For $|V|$>1.5V we generally observe a rapid increase in conductance (Supplementary Figure 3b) and a redshift of the coupled mode (Supplementary Figure 3a), possibly due to disruption of the molecular layer and formation of metallic conductive links across the junction that increase conductance and short the coupled plasmonic mode. This redshift is only rarely reversible and suggests a morphological change in the junction structure.

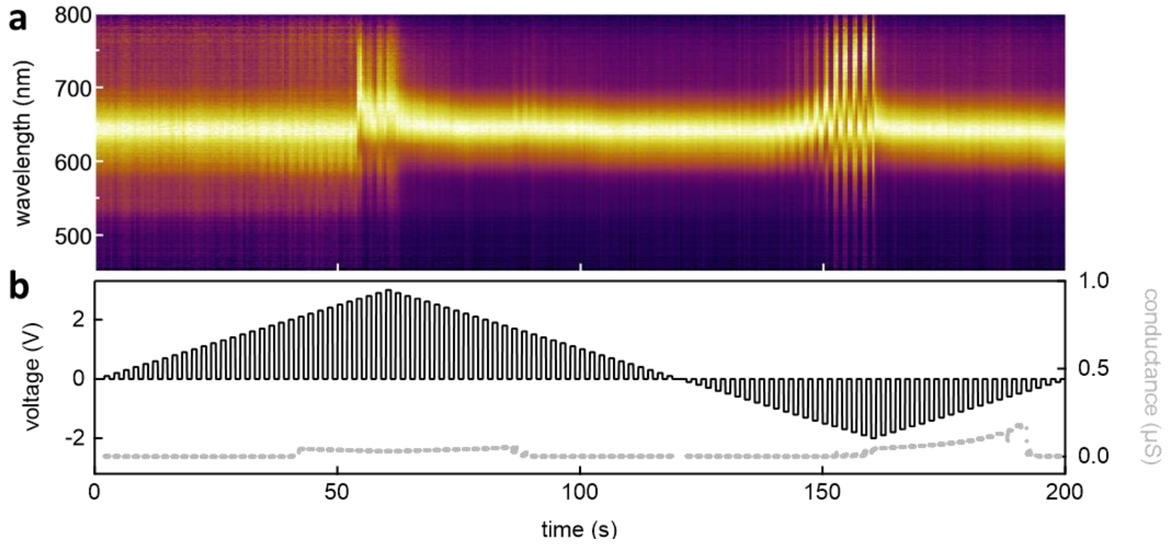

Supplementary Figure 3 | In-situ darkfield scattering spectroscopy of BPDT junctions. **a**, AuNP is monitored with darkfield spectroscopy in real time (scaled at each time point) while **b**, electrical measurements are recorded on the AuNP molecular junction. Coupled mode amplitude and position are stable for $|V|$<1.5V.

Supplementary Note 3. In-situ Raman spectroscopy

Raman spectroscopy is performed in real time on molecules inside a single AuNP junction. The plasmonic enhancement of optical field in the junction gap amplifies light-matter interactions enabling surface enhanced Raman spectroscopy (SERS) with a typical signal of >10^5counts/mW/s for the molecular vibrational signatures, allowing real time spectroscopy with <1s integration times. Electrical measurements are performed at a faster rate of 50 datapoints/s given by the Keithley SMU, so tens of I-V data points are collected for each spectrum acquisition.

Reduction in the Raman intensity for voltages above 0.5V are consistently observed for BPDT in more than 70% of the single AuNP junctions contacted with the cantilever (Supplementary Figure 4a,b). The remaining cases are accounted for by variations in AuNP size or local imperfections in the parylene insulating layer leading to poor contact with the AuNP crown. *In-situ* spectroscopic and electrical measurements on a particular junction in the $|V|$<1.5V regime can typically be cycled for tens of minutes before mechanical vibrations of the cantilever cause local displacement of the AuNP leading to permanent modifications.

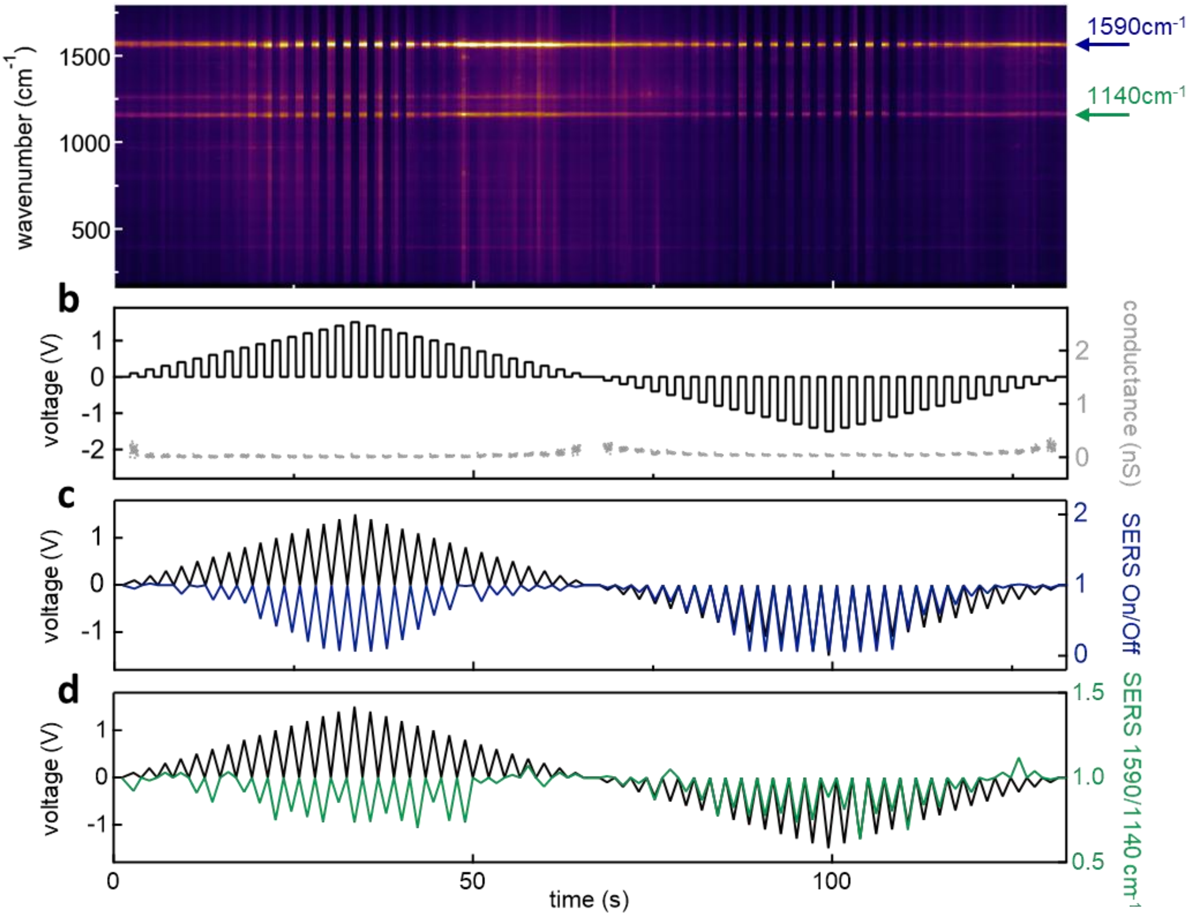

Supplementary Figure 4 | In-situ Raman spectroscopy of BPDT junctions. **a**,**b** In-situ Raman spectroscopy of a single AuNP junction with BPDT molecular layer. **c**, Corresponding voltage (black) and SERS intensity ratio (blue) calculated as SERS(V)/SERS(V=0) for the 1590cm^{-1} ring-ring CC stretching mode. Each data point is the average of consecutive measurements taken at the same voltage. **d**, Ratio of amplitudes for peaks at 1590cm^{-1}/1140cm^{-1}, showing that modulation is stronger for the 1590cm^{-1} peak corresponding to the inter-ring CC bond.

We observe a decrease of up to 95% in the SERS intensity for the Raman signal of BPDT for $|V|$>1V (Fig.S4c). The effect is first visible around |V|=0.5V, saturating for $|V|$>1V, and is symmetric for positive/negative voltages in most experimental realisations. The SERS peak near 1590cm^{-1}, corresponding to the stretching of the central CC inter-ring bond, shows a stronger modulation than the other peaks in the 1100-1250cm^{-1} region (Supplementary Figure 4d). This highlights that this bond is more affected by the applied voltage due to the twist between the rings, and is in good agreement with the same trend observed in DFT calculations. We also observe a decrease of the SERS background by up to a factor 2 for $|V|$>1V, suggesting a $2^{1/4}$-1=16% decrease in the electromagnetic field intensity in the gap (since SERS intensity scales as E^4 in the NPoM gap[2]). This could be due to a reduction in gap refractive index at optical frequencies, however this would produce blueshifts in DF while redshifts are seen experimentally (but at higher voltages where current flows).

DFT calculations (Figure 3 main text) predict a small redshift of a few cm^{-1} as the twist angle between the BPDT rings is increased from 0 to 90°. This shift is not experimentally observed in our measurements nor in previous reports[3,4], suggesting this is due to inaccuracies in the DFT model at

this order. DFT calculations also involve a single molecule attached to individual Au atoms, whereas a real device includes extended Au electrodes and close-packed molecules.

In-situ SERS of 2-naphtalenethiol in our AuNP junctions shows no changes of peak intensity correlated with voltage for any of the molecular vibration peaks (Supplementary Figure 5). Variations in the signal are compatible with minor intensity fluctuations generally observed in all measurements. The 2-naphtalenethiol molecule still has two rings, but these are locked in the same plane since they share two carbon atoms, so they are unable to twist like the biphenyl molecules and thus do not produce the same modulation in SERS intensity.

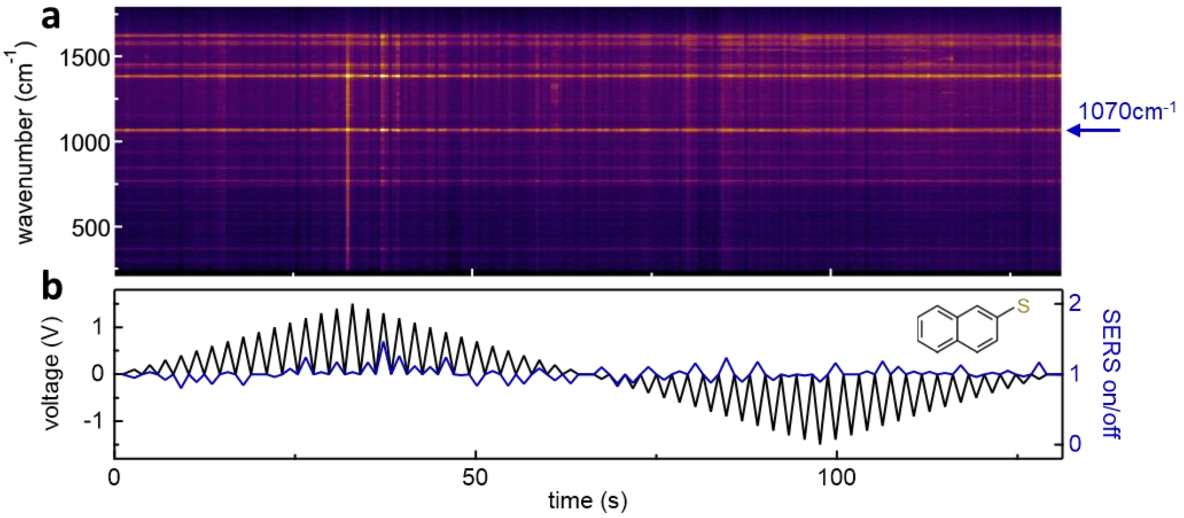

Supplementary Figure 5 | SERS of 2-naphtalenethiol. **a**, SERS signal from an AuNP junction fabricated with 2-naphtalenethiol. **b**, Corresponding voltage applied across the junction (black) and SERS intensity of the 1070cm^{-1} peak (blue) calculated as SERS(V)/SERS(V=0). Each data point is the average of consecutive measurements taken at the same voltage.

Different functional groups at the ends of the biphenyl ring structure affect the conductance of the AuNP junction near the molecule-electrode interfaces, producing different effects in the capacitive-molecular energy balance discussed in the main text. For BPDT we observe the maximum level of modulation, with a symmetric decrease in SERS with voltage as high as 95% (Supplementary Figure 6a). BMMBP shows a modulation of up to 75%, but stronger for negative voltages than for positive ones (Supplementary Figure 6b). Even though the BMMBP molecule itself is also symmetric, the additional bonds at its ends compared to BPDT can introduce variations in the SAM packing during assembly on the substrate or AuNP, causing the observed some asymmetry in SERS modulation. Finally, for BPT and CN-BPT we observe a symmetric SERS change of up to 30%, with the modulation occurring only at $|V|$>2V for CN-BPT.

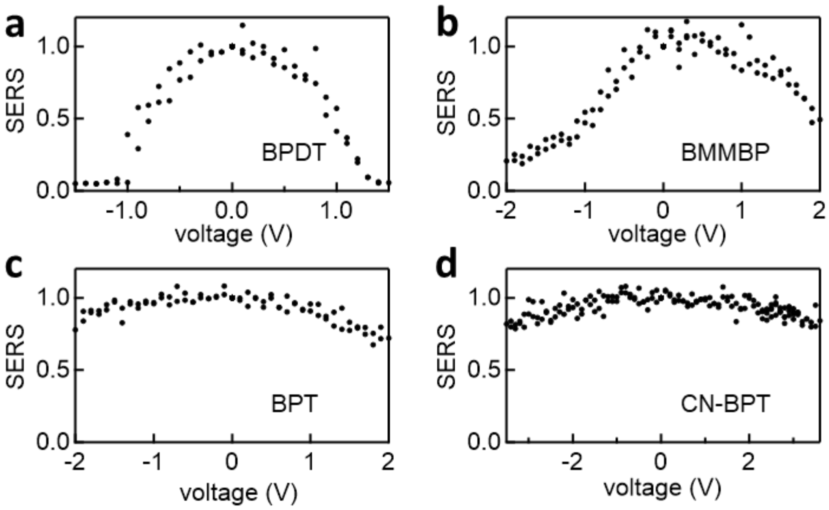

Supplementary Figure 6 | Role of functional groups in SERS modulation. Change in SERS intensity as a function of voltage for **a**, BPDT, **b**, BMMBP, **c**, BPT, and **d**, CN-BPT.

Supplementary Note 4. Electrical characteristics

NPoM junctions are characterised using a source-measure unit (Keithley SMU, see Methods main text) in 4-wire configuration. To ensure that the cantilever contacting method applied to NPoM junctions does not affect the measured values of molecular conductance, we separately verify the electrical performance of the cantilever-NPoM system.

The conductance of the cantilever itself is measured by electrically contacting a set of Au wires of varying width patterned directly on SiO_2 with e-beam lithography (Supplementary Figure 7a). Each wire is contacted from the top in a direction parallel to the cantilever. For the smallest Au wire of width 50nm we measure a linear ohmic response with conductance G=30G_0 (Supplementary Figure 7b), while for 100nm wide wires we measure 80G_0. For wider wires up to 50µm we always measure a conductance of 80G_0, indicating that this is the conductance limit of the cantilever contacting method, set by the conductive coating on the cantilever.

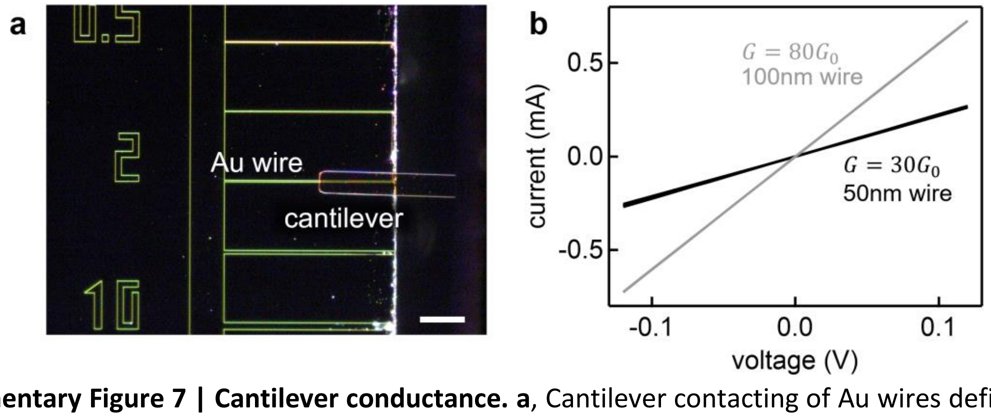

Supplementary Figure 7 | Cantilever conductance. a, Cantilever contacting of Au wires defined with e-beam lithography; scale bar is 50µm. **b**, Current-voltage characteristic of cantilever-wire contact is linear with G=30G_0 for 50nm wide wires, and G=80G_0 for ≥100nm wires.

The conductance of the NPoM system contacted with the cantilever is measured by fabricating a sample with the same geometry described in the main text but no molecular SAM in the NPoM gap, with the AuNP thus directly contacting the bottom Au mirror. In these single particle devices we consistently measure a linear ohmic I-V response with conductance G=13G_0=10^{-3}S (Supplementary

Figure 8). Conduction occurs through the AuNP, but a simple conductance calculation from the nominal geometry of a NP (20nm wide facet, 100nm diameter, Au resistivity $2.2\times10^{-8}\Omega$m) predicts a conductance of ≈0.1S. This suggests that conduction actually happens through a smaller contact area, likely defined by the local geometry of the NP facet in the gap and roughness of Au mirror. From previous investigations of conductance in nm-sized Au constrictions[5] and given that the bottom Au mirror and Au coating on the cantilever are deposited in the same conditions, we infer that conduction in our system is limited by two equal contact points with diameter 4nm, respectively at the cantilever-AuNP and AuNP-mirror interfaces. In presence of a SAM, we thus estimate that ≈50-60 molecules within this contact region determine the electrical properties of our devices.

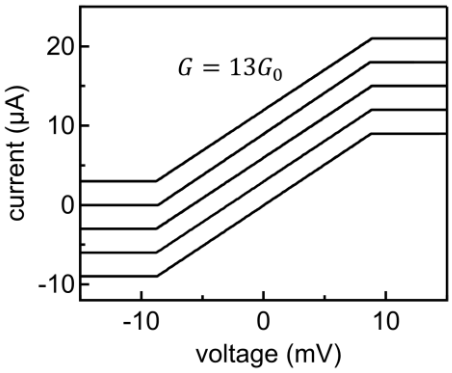

Supplementary Figure 8 | Conductance across bare AuNP. Cantilever contacting of single NPoMs without SAM shows linear I-V response with conductance G=13G_0. Each trace corresponds to a different single AuNP junction. Traces in the plot are each offset by 3μA, all use 9μA compliance current limit.

Adding a SAM of BPDT to the NPoM system immediately drops the junction conductance (Supplementary Figure 9a), with measured low-bias conductance values across different junctions ranging from $1.5\times10^{-6}G_0$ to $1\times10^{-5}G_0$ per NP (Supplementary Figure 9b). This is >5 orders of magnitude smaller than the conductance across a single AuNP without SAM, and we thus conclude that the cantilever contacting technique with AuNPs has a negligible effect on the measurements of molecular conductance. We attribute the observed variability in the conductance across different junctions to variations in the number of contacted molecules due to surface roughness and NP facet size, but note that most junctions show conductance in the $10^{-6}G_0<G<10^{-5}G_0$ range (Supplementary Figure 9c).

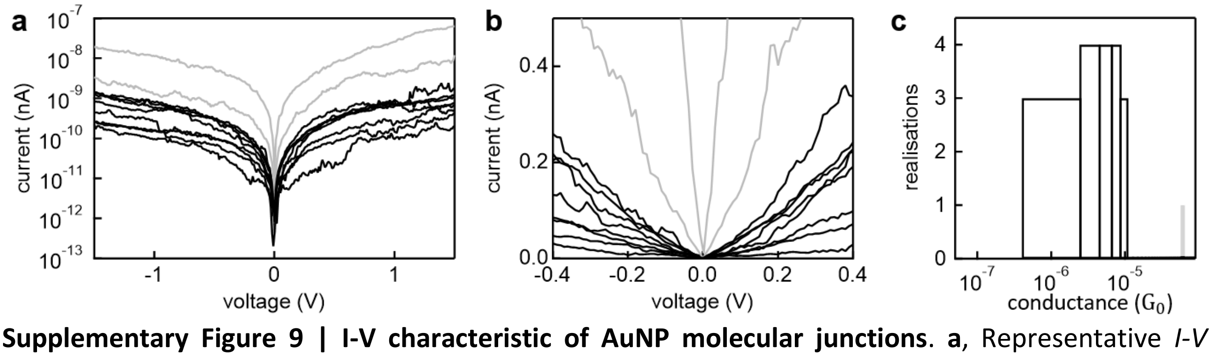

Supplementary Figure 9 | I-V characteristic of AuNP molecular junctions. **a**, Representative *I-V* characteristic of 10 AuNP junctions for the case of a BPDT SAM. Black curves are for single NP junctions, grey for junctions with 2 NPs. **b**, Detail of (a) in linear scale in low bias regime, showing linear response typical of direct tunnelling. **c**, Histogram of low-bias (|V|<0.2V) conductance values from data in b, normalised to single NP.

Supplementary Note 5. Single molecule twist switching

As noted in the main text, the 20nm wide facets of 100nm AuNP nanogaps accommodate ~100 molecules in a single junction However an effect explored in the last few years [see main text ref[17]] allows single molecules SERS to be tracked in real time in the same nanocavities. Strong enough optical fields in the NPoM gap can transiently pull out atoms from the facet surface, which further enhances light confinement and produces fleeting SERS lines in addition to the normal Raman. We detect these 'picocavity' events (Supplementary Figure 10a), revealing again the same SERS intensity modulation on the lines which are shifted by molecular-interactions with the Au adatom (Supplementary Figure 10b). In particular the voltage-induced suppression of single-molecule SERS matches that of the main molecular SERS peaks (dashed box). This shows that the same voltage-induced twist model applies to single-molecule junctions (Supplementary Figure 10b). Bias is not found to modulate these Au adatoms in different ways, nor does the modified Au-molecule interaction modify the twist forces dramatically. Generally all picocavities observed show the same twist switching, matching that of the main SERS peaks.

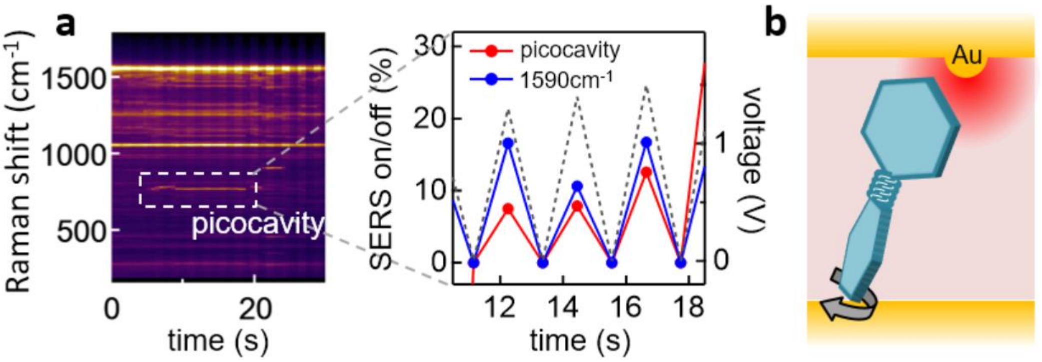

Supplementary Figure 10 | Single molecule switching from picocavities. **a**, Single molecule switching for BPT showing transient picocavities observed in SERS spectra (dashed box). **b**, Enhanced optical field in vicinity of protruding Au adatom inside nanogap, enhances SERS to single molecule level.

Supplementary Note 6. Structures for DFT calculations

DFT calculations without bias are performed as described in the Methods in the main text[6–12]. A typical molecular structure is shown in Supplementary Figure 11, with a BPDT molecule anchored to a Au atom at each thiol terminal group. In the calculations the dihedral angle is increased from 0 to 90°.

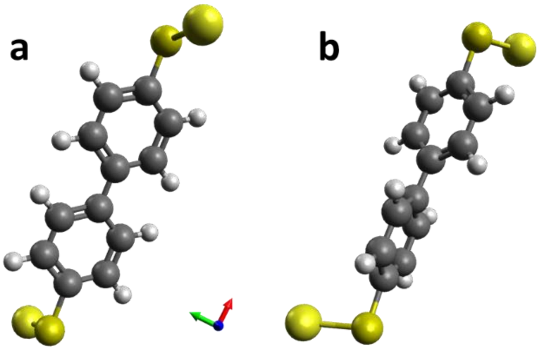

Supplementary Figure 11 | DFT structures. **a,b**, BPDT molecule used in DFT calculations anchored at two Au atoms shown at (a) 0° and (b) 65° twist.

Supplementary Note 7. Thermal excitation of BPDT twist

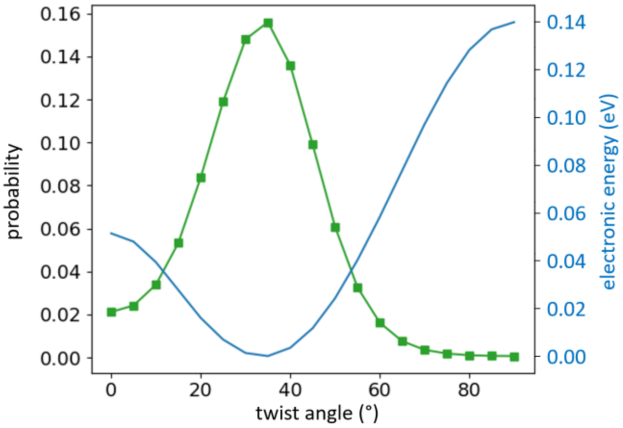

Supplementary Figure 12 | Boltzmann populations from DFT calculations of twist in BPDT at room temperature. BPDT molecule used in DFT as in Supplementary Figure 11. The probability for each twist angle is extracted, as well as the potential at each twist. This clearly shows the measurement of twist gives a clear angular position, around 35±10°.

Supplementary Note 8. Strain induced changes to DFT twist

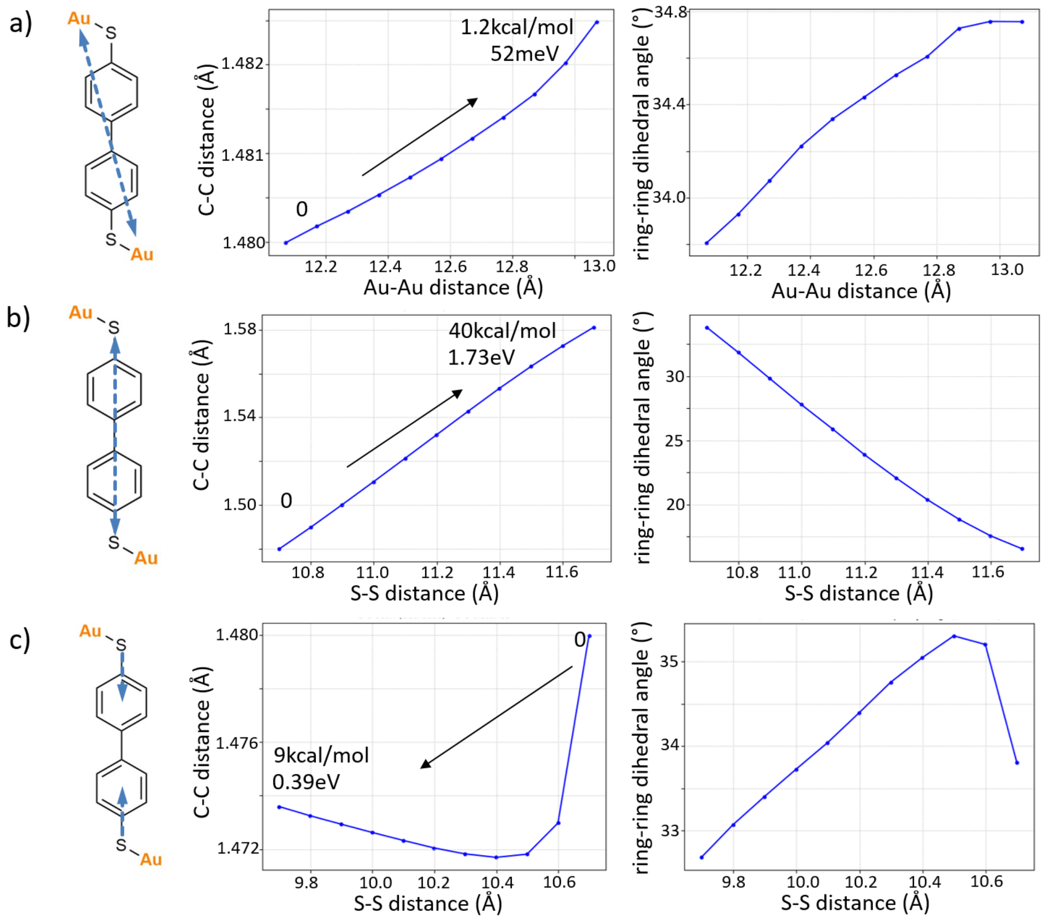

Supplementary Figure 13 | Effect of applying strain on BPDT from DFT. a, Stretching the Au-Au distance from 12.07 to 13.07Å requires relatively small energy, but barely changes the twist angle. **b**, Stretching the S-S distance from 10.70 to 11.70Å requires very large energies and so is not possible. **c**,

Similarly compressing the S-S distance from 10.70 to 9.70Å bends the molecule requiring large energies.

Supplementary Note 9. Parameters for electrical model of BPDT in nano-junction

We use the model in the main text, with two molecule-metal junctions (capacitance $C_{1,3}$ and conductance $G_{1,3}$) for the back-to-back Schottky diodes, and a junction between the two conducting aryl rings in the centre of the molecule with capacitance C_2 and conductance G_2. Because a set of similar molecules with different twist angles have already been measured experimentally, these are not free parameters.

Conductive AFM and STM break-junction experiments in liquid indicate that the conductance G_2 through biphenyl molecules is controlled by the inter-ring twist, $G_2 = G_{CC}(1 + g\cos^2\theta)$ where G_{CC}~76 µS when the rings are exactly orthogonal so there is no $\pi - \pi$ overlap (see refs [23-26] in main text). The value of g is then set from the total conductance when the rings are planar (note that what is measured in STM is the total conductance and not just the molecular conductance), using $G_t(\theta = 0)$~1.5 mS (ref [26] in main text) which implies G_1~ 2.4 mS, and that g~50. This is indeed what prior experiments quantitatively show, that the ring-ring conduction increases by a factor of 50 when the ring twist angle approaches zero.

For the capacitance of these junctions, we calculate $C = q/V$ using the charge distribution and potential profile obtained from DFT modelling of the junction under bias (see Supplementary Note 11), and obtain C_2=0.015aF and C_1=0.04aF. These values are reasonably close to simple parallel-plate estimates: $C_2 = \varepsilon_0\varepsilon_r A_{CC}/d_{CC}$ ~ 0.012aF with ε_r=2,[13] $A_{CC} = \pi(0.15\text{nm})^2$=0.07nm^2, and the frontier orbital separation of the carbons between the rings d_{CC}~ 1Å. The contact capacitance $C_1 = C_3 = \eta\varepsilon_0\varepsilon_r A/d_{S-Au}$ ~ 1.8aF, where the factor η comes from the capacitance of the sphere-plate system, $\eta = \ln\left(1 + \frac{D\pi^2}{4d_{S-Au}}\right)$ ~4.3[14], and A=0.23nm^2/molecule from the packing of the SAM, with the frontier orbital separation of the Au-S estimated from DFT as d_{S-Au}~ 0.1Å. This suggests that the DFT values for 2D Au contacts are compatible with a real 3D junction given that it has a much larger contact area. We find that an adjusted value of C_1=1.5aF provides the best fit of the model to experimental data, and gives the fit described in the main text and Fig.4b,c. Indeed the junction capacitance from molecule - metal contact increases, because of the larger charge screening possible for the 3D Au contact geometry. The controlling parameter is the ratio $\sqrt{C_1/C_2}$~15, which can be intuitively thought as representing how much wider the contact size for the metal-molecule contact is than the C-C link between the rings inside the molecule.

For the BMMBP, we can estimate the reduction in conductivity along the chain from its well characterised effect on tunnelling.[15,16] A universal scaling is found when inserting an additional carbon in the chain, with β~0.9. Using this in the model shows that the change in SERS at bias of 1V reduces as this molecular conductivity reduces (Supplementary Figure 14a).

For both BPT and BPTCN, the top contact conductance and capacitance are expected to decrease. Again using this in the model, including both V_1,C_1 and V_3,C_3 given the molecular asymmetry, shows that the data would be explained for capacitances more than 30x times smaller than the BPDT contact capacitance where the thiol binds directly to the Au (Supplementary Figure 14b).

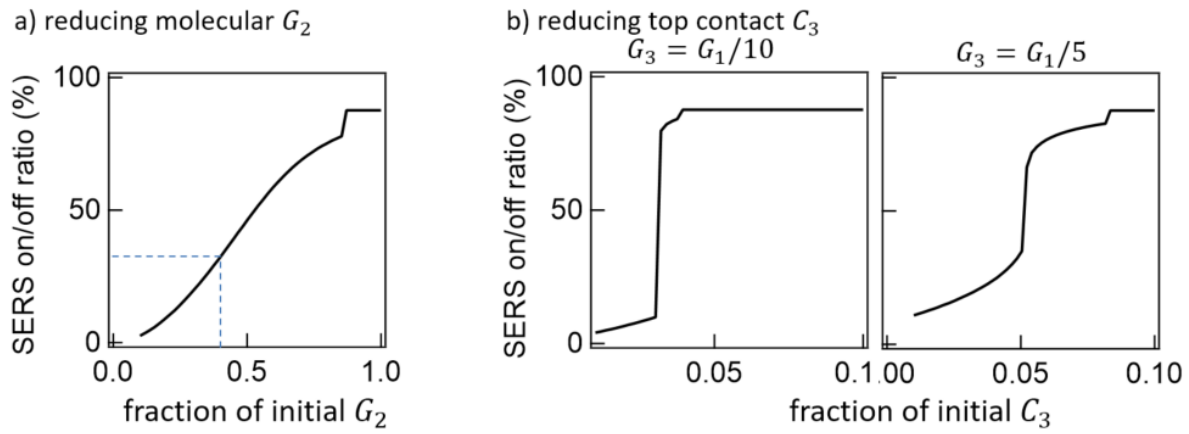

Supplementary Figure 14 | Effect of changes to circuit model in voltage-dependent suppression of SERS. a, Keeping all parameters the same as above, except reducing the molecular conductance G_2. This shows that suppression reduces as molecular conductance reduces. Dashed lines show estimate for BMMBP. **b**, For several values of reduced top contact G_3, shows SERS scaling with reduced contact capacitance C_3.

Supplementary Note 10. DFT calculations of charged BPDT molecules

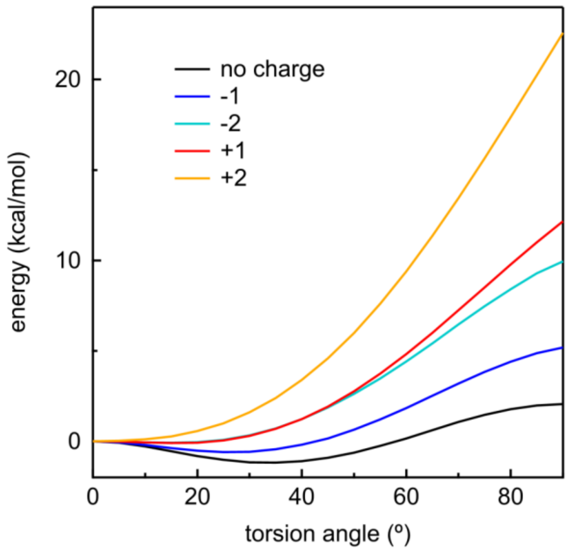

Supplementary Figure 15 | Energy potential of charged BPDT molecules vs ring torsion angle. DFT calculations on BPDT (Supplementary Note 6, gas phase) predict an energy minimum for a ring torsion angle of 35° without molecular charging. Adding net charge to the molecule, either positive or negative, induces a shift of the equilibrium position to smaller angles and gives an increase in Raman cross section in agreement with previous findings[3,4]. On the contrary, the observed decrease in Raman signal with bias in our experiments, indicates that molecular charging cannot be responsible.

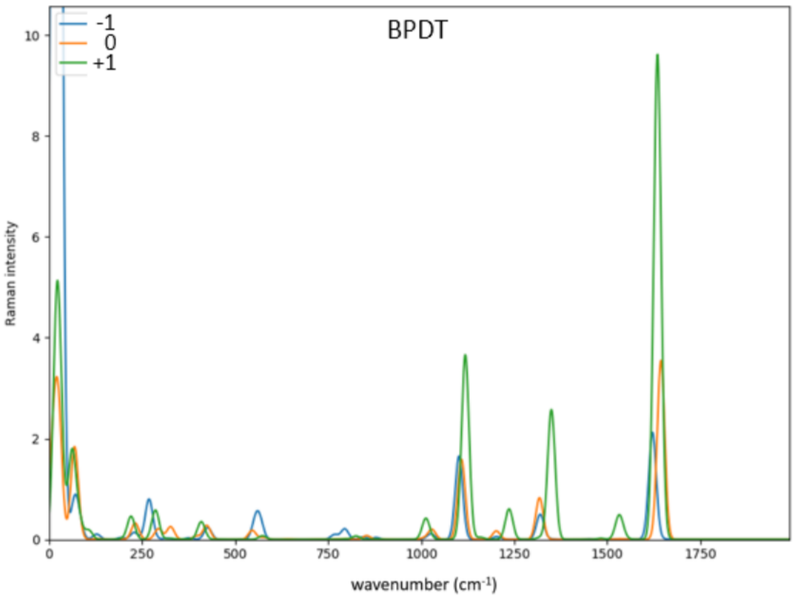

Supplementary Figure 16 | Raman spectra of charged BPDT. DFT calculations of BPDT indicate that positive (green, +1) or negative (blue, -1) molecular charge induces shifts in the Raman peak positions from those of the neutral molecule (orange). No shifts are observed experimentally when bias is applied across NPoM junctions.

Supplementary Note 11. DFT modelling of BPDT junction under non-equilibrium condition

A single molecule BPDT junction is first modelled with a 3D lattice of Au atoms in the leads (Supplementary Figure 17a), using double-zeta polarized basis set to verify consistency with the simplified 2D lattice, and with single-zeta basis set adopted later. This is to avoid computationally extremely expensive calculations using the 3D lattice of Au atoms in the leads. The energy profile shows qualitative agreement between the two models (Supplementary Figure 17). The simpler model with a 2D lattice described in the main text provides results consistent with the 3D lattice system and allows calculation of potentials and transport properties for various bias voltages including at higher bias voltages. Our calculations using this model show that the minimum energy configuration at zero bias is $\theta \sim 20°$, which shifts to higher angles as the bias in increased (Supplementary Figure 18).

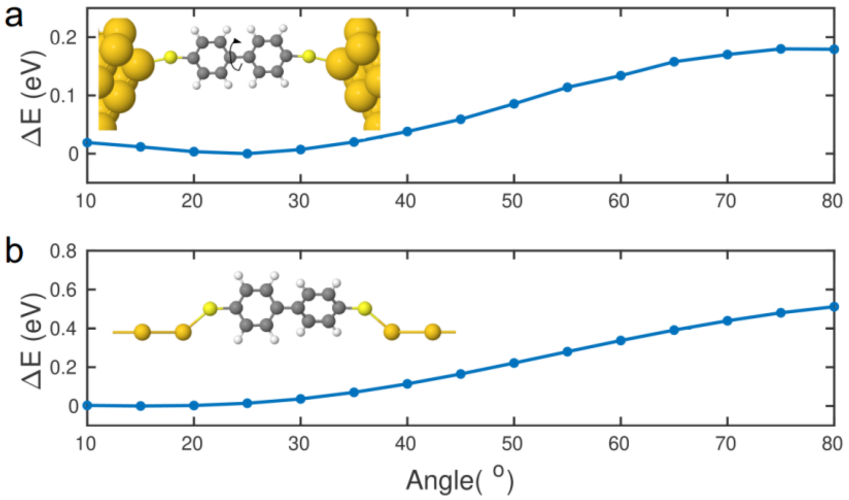

Supplementary Figure 17 | DFT calculations of total energy with different electrode size. Single molecule BPDT junction modelled with **a**, a 3D lattice of Au atoms and **b,** a 2D lattice of Au atoms in the leads, as the angle between rings is varied.

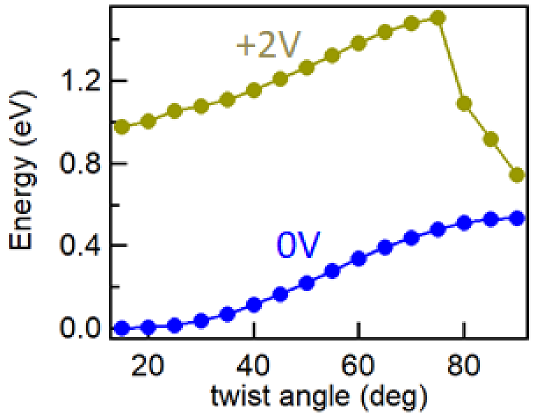

Supplementary Figure 18 | DFT calculations under bias for 2D Au lattice. Minimum energy configuration in 2D lattice model shifts from $\theta\sim30°$ to $\theta\sim90°$ as bias is increased from V_b=0V to V_b=2V.

Furthermore, it is found that the induced potential and charge fluctuations swap sides when the applied bias is reversed (Supplementary Figure 19). This confirms that one contact acts as rectifying while the other is conducting, as expected for back-to-back Schottky diodes (discussed in the main text).

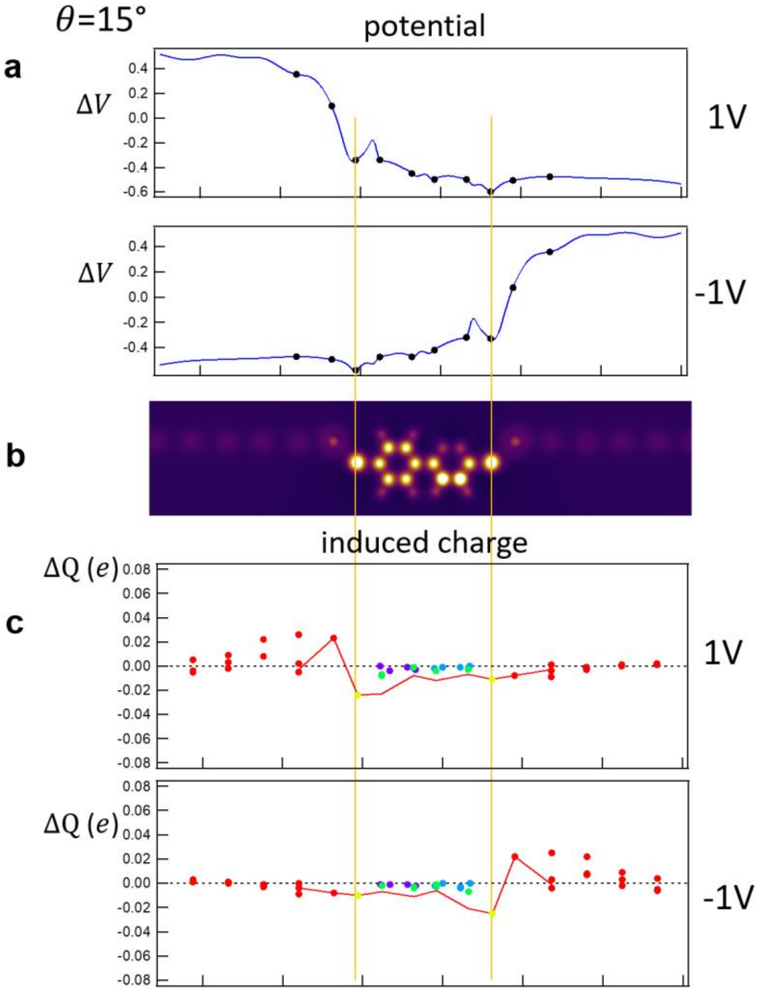

Supplementary Figure 19 | Potential profile and charge distributions in 2D lattice model, for θ=15°. **a**, Under 1V bias, the induced potential drops almost entirely at the S-Au interface of the metal lead

with the higher electron potential (negative contact). The profile is mirrored when the bias polarity is inverted. **b**, Potential map. **c,** Charge distribution in units of elementary charge for each atom in the model (red: Au, yellow: S, green: C, purple/blue: H). Positive charge accumulates at the link-Au atom and neighbouring Au atoms, while negative charge is concentrated on the S atom, inducing a strong dipole at the molecule-lead interface, causing the large potential drop.

To calculate transport properties of the junctions with different twist angle and bias voltages, from the converged mean-field Hamiltonian $H(V_b)$ obtained from DFT calculation, we calculated transmission coefficient $T(E, V_b)$ of electrons with energy E passing from one electrode to the other[17,18] via the relation $T(E) = Tr(\Gamma_L\ (E)G^R(E)\Gamma_R\ (E)G^{R\dagger}(E))$ where $\Gamma_{L,R}(E) = i(\sum_{L,R}(E) - \sum_{L,R}{}^{\dagger}(E))$ describes the level broadening due to the coupling between left L and right R electrodes and the central scattering region, $\Sigma_{L,R}(E)$ are the retarded self-energies associated with this coupling and $G^R\ = (ES - H - \sum_L^e - \sum_R^e)^{-1}$ is the retarded Green's function, where H is the Hamiltonian and S is the overlap matrix obtained from *SIESTA* implementation of DFT under non-equilibrium conditions[19]. We then calculate the room-temperature current $I(V_b)$ from obtained $T(E, V_b)$ using Landauer formula $I(V_b) = \frac{2e}{h}\int_{-\infty}^{+\infty} dE\ T(E, V_b)\left(f\left(E, T, \frac{V_b}{2}\right) - f\left(E, T, -\frac{V_b}{2}\right)\right)$. The measured current is an ensemble average of a few tens of molecules in the junction. Each molecule junction has slightly different binding to the electrodes and therefore the alignment of frontier orbitals relative to the electrode Fermi energy can be different from one junction to the next. In order to account for this effect, we calculate $I - V$ characteristics using a range of electrodes Fermi energy ([-0.25, 0.25] eV) as shown in Supplementary Figure 20a. Supplementary Figure 20b shows the average current calculated from $I - V$ curves in Supplementary Figure 20a. The corresponding electrical conductance is shown in Supplementary Figure 20c. Clearly, there is a smooth crossover from currents through the 15°-twist angle (lowest energy configuration at low bias $V < V_t$) to that of 90°-twist angle (lowest energy configuration at higher bias $V\ > V_t$).

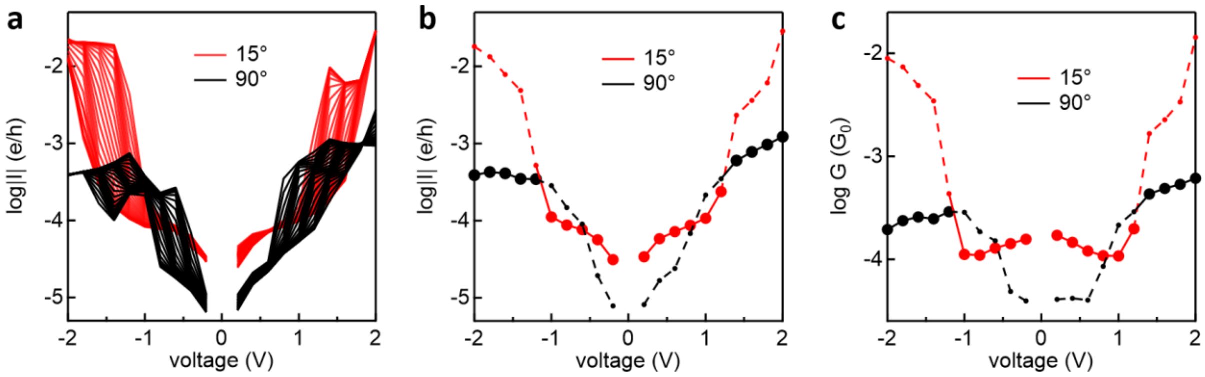

Supplementary Figure 20 | Transport in BPDT junctions. **a**, Calculated current though a single molecule BPDT junction as a function of applied bias. Small variations (±0.25eV fluctuation range plotted) in the Fermi energy change the transport properties for both θ=15° and θ=90°. **b**, Average of curves plotted in (a), together with **c**, corresponding conductance. In an NPoM junction with many molecules, switching towards θ=90° likely occurs at slightly different voltages for each molecule, so no sharp change is conductance is expected around the transition voltage.

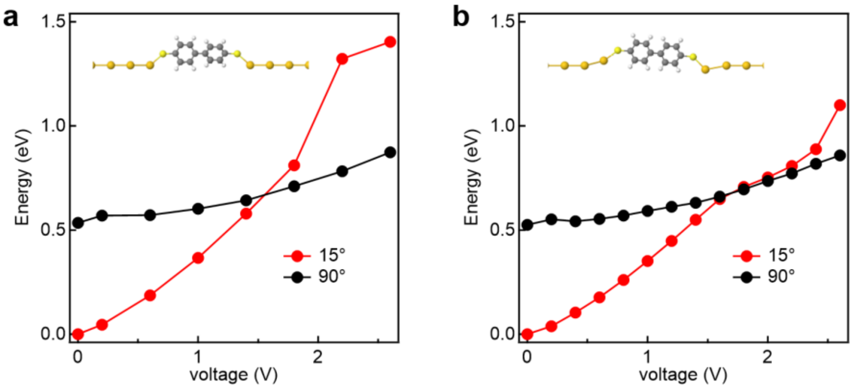

Supplementary Figure 21 | Effect of binding geometry on transport calculations. Configuration energies obtained from DFT calculations under bias for flat Au electrodes (**a**), and displaced link-Au atoms (**b**). The specific atomic geometry of the molecule-electrode interface affects the configuration energy profile, but does not qualitatively change the ring twist behaviour.

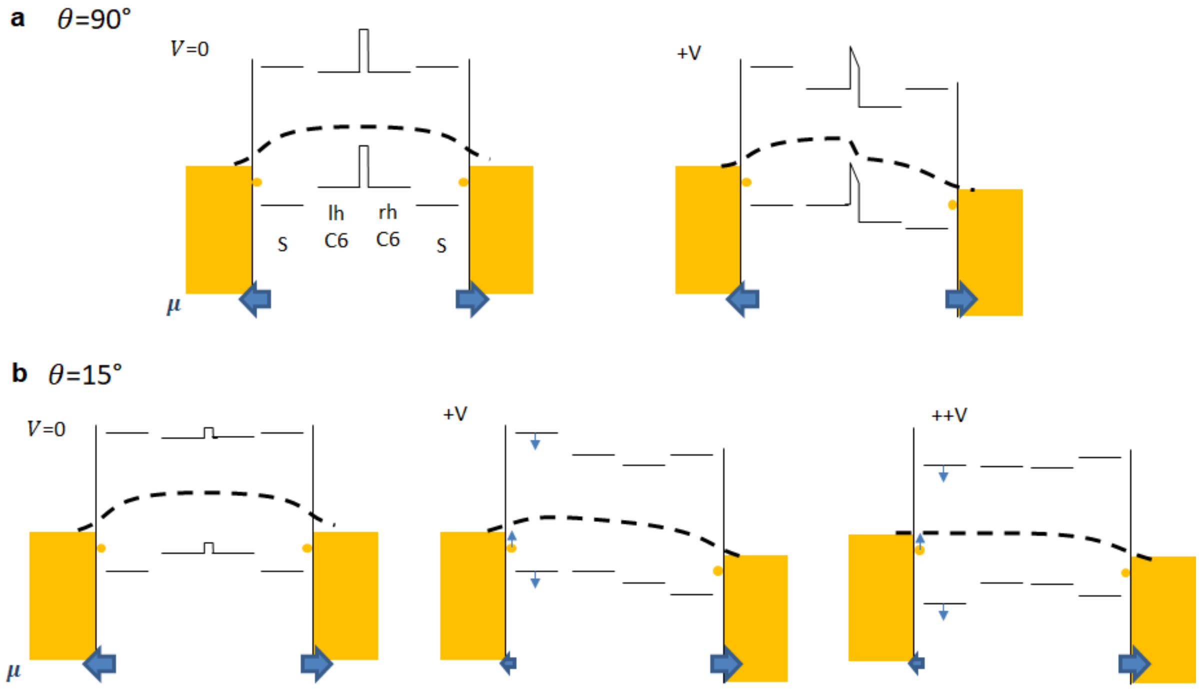

Supplementary Figure 22 | Simple tunnelling barrier picture of transport during molecular twisting. Initially upon SAM formation, electron transfer from Au to S creates a Schottky barrier at each electrode (blue arrows), with quasi-Fermi level shown dashed. **a**, At θ=90° the central CC bond presents a high barrier, which dominates transport. Hence for voltage applied the potential drops mostly across the centre of the molecule. **b**, At θ=15° there is no barrier in the centre of the molecule, so the only barriers are those set by the contact dipoles. Since the electron flux is proportional to $v|\psi|^2$ (with v the flux velocity) and is conserved, regions with higher barrier give smaller v ($v \propto k \propto 1/\sqrt{2mE}$) and thus a larger wavefunction amplitude. This means that applying a positive bias to the right hand electrode (decreasing electron potential there) builds up electrons on the link-Au on the left side. This reduces the lhs surface dipole, decreasing the tunnel barrier which allows current to flow. The dipole on the rhs of the molecule is unchanged because there is no charge build-up (Fig. 4d

red in main text). Note also that if the bias direction is reversed, the same contact dipole is modified on the negative electrode on the rhs (Supplementary Figure 19a).

Supplementary References

1. Noordegraaf, J. Conformal coating using parylene polymers. *Med Device Technol* **8**, 14–20 (1997).
2. Baumberg, J. J., Aizpurua, J., Mikkelsen, M. H. & Smith, D. R. Extreme nanophotonics from ultrathin metallic gaps. *Nat. Mater.* **18**, 668 (2019).
3. Bi, H. *et al.* Voltage-Driven Conformational Switching with Distinct Raman Signature in a Single-Molecule Junction. *J. Am. Chem. Soc.* **140**, 4835–4840 (2018).
4. Cui, L. *et al.* In Situ Gap-Mode Raman Spectroscopy on Single-Crystal Au(100) Electrodes: Tuning the Torsion Angle of 4,4′-Biphenyldithiols by an Electrochemical Gate Field. *J. Am. Chem. Soc.* **133**, 7332–7335 (2011).
5. Strachan, D. R. *et al.* Real-Time TEM Imaging of the Formation of Crystalline Nanoscale Gaps. *Phys. Rev. Lett.* **100**, 056805 (2008).
6. Becke, A. D. Density-functional thermochemistry. III. The role of exact exchange. *J. Chem. Phys.* **98**, 5648–5652 (1993).
7. Grimme, S., Ehrlich, S. & Goerigk, L. Effect of the damping function in dispersion corrected density functional theory. *J. Comput Chem.* **32**, 1456–1465 (2011).
8. Weigend, F. & Ahlrichs, R. Balanced basis sets of split valence, triple zeta valence and quadruple zeta valence quality for H to Rn: Design and assessment of accuracy. *Phys. Chem. Chem. Phys.* **7**, 3297–3305 (2005).
9. Polavarapu, P. L. Ab initio vibrational Raman and Raman optical activity spectra. *J. Phys. Chem.* **94**, 8106–8112 (1990).
10. Krishnakumar, V., Keresztury, G., Sundius, T. & Ramasamy, R. Simulation of IR and Raman spectra based on scaled DFT force fields: A case study of 2-(methylthio)benzonitrile, with emphasis on band assignment. *J. MOLEC. STRUCT.* **702**, 9–21 (2004).
11. Michalska, D. & Wysokiński, R. The prediction of Raman spectra of platinum(II) anticancer drugs by density functional theory. *Chem. Phys. Lett.* **403**, 211–217 (2005).
12. Gaussian 09 Citation | Gaussian.com. https://gaussian.com/g09citation/.
13. Romaner, L., Heimel, G., Ambrosch-Draxl, C. & Zojer, E. The Dielectric Constant of Self-Assembled Monolayers. *Adv. Funct. Mater.* **18**, 3999–4006 (2008).
14. Benz, F. *et al.* Generalized circuit model for coupled plasmonic systems. *Opt. Express, OE* **23**, 33255–33269 (2015).
15. Prodan, E. & Car, R. Tunneling Conductance of Amine-Linked Alkyl Chains. *Nano Lett.* **8**, 1771–1777 (2008).
16. Wang, G., Kim, T.-W., Jang, Y. H. & Lee, T. Effects of Metal−Molecule Contact and Molecular Structure on Molecular Electronic Conduction in Nonresonant Tunneling Regime: Alkyl versus Conjugated Molecules. *J. Phys. Chem. C* **112**, 13010–13016 (2008).
17. Sadeghi, H. Theory of electron, phonon and spin transport in nanoscale quantum devices. *Nanotechnology* **29**, 373001 (2018).
18. Ferrer, J. *et al.* GOLLUM: a next-generation simulation tool for electron, thermal and spin transport. *New J. Phys.* **16**, 093029 (2014).
19. Soler, J. M. *et al.* The SIESTA method for ab initio order-N materials simulation. *J. Phys.: Condens. Matter* **14**, 2745–2779 (2002).
